# Alternative lengthening of telomeres in molecular subgroups of paediatric high-grade glioma

**DOI:** 10.1007/s00381-020-04933-8

**Published:** 2020-10-31

**Authors:** Simone Minasi, Caterina Baldi, Francesca Gianno, Manila Antonelli, Anna Maria Buccoliero, Torsten Pietsch, Maura Massimino, Francesca Romana Buttarelli

**Affiliations:** 1 Department of Radiological, Oncological and Anatomo-Pathological Sciences, Sapienza University of Rome, Rome, Italy; 2 Department of Human Neurosciences, Sapienza University of Rome, Rome, Italy; 3 Department of Molecular Medicine, Sapienza University of Rome, Rome, Italy; 4 Pathology Unit, Meyer Children’s University Hospital, I-50139 Florence, Italy; 5 Institute of Neuropathology, DGNN Brain Tumour Reference Centre, University of Bonn Medical Centre, Bonn, Germany; 6 Paediatric Unit, Fondazione IRCCS Istituto Nazionale dei Tumori, Milano, Italy

**Keywords:** Telomeres, Alternative lengthening of telomeres, H3.3, Paediatric high-grade gliomas, pHGG

## Abstract

**Purpose:**

The maintenance of telomere length prevents cancer cell senescence and occurs via two mutually exclusive mechanisms: (a) reactivation of telomerase expression and (b) activation of alternative lengthening of telomeres (ALT). ALT is frequently related to alterations on ATRX, a chromatin-remodelling protein. Recent data have identified different molecular subgroups of paediatric high-grade glioma (pHGG) with mutations of *H3F3A*, *TERTp* and *ATRX*; however, differences in telomere length among these molecular subgroups were not thoroughly examined.

**Methods:**

We investigated which genetic alterations trigger the ALT mechanism in 52 IDH-wildtype, 1p/19q-wildtype pHGG. Samples were analysed for telomere length using Tel-FISH. ATRX nuclear loss of expression was assessed by IHC, *H3F3A* and *TERTp* mutations by DNA sequencing, and *TERTp* methylation by MS-PCR.

**Results:**

Mutant H3.3 was found in 21 cases (40.3%): 19.2% with K27M mutation and 21.1% with G34R mutation. All H3.3G34R-mutated cases showed the ALT phenotype (100%); on the opposite, only 40% of the H3.3K27M-mutated showed ALT activation. ATRX nuclear loss was seen in 16 cases (30.7%), associated sometimes with the G34R mutation, and never with the K27M mutation. ATRX nuclear loss was always related to telomere elongation. *TERTp* C250T mutations were rare (5.4%) and were not associated with high intensity Tel-FISH signals, as *TERTp* hyper-methylation detected in 21% of the cases. H3.3/ATRX/*TERTp*-wildtype pHGG revealed all basal levels of telomere length.

**Conclusion:**

Our results show a strong association between H3.3 mutations and ALT, and highlight the different telomeric profiles in histone-defined subgroups: H3.3-G34R mutants always trigger ALT to maintain telomere length, irrespective of ATRX status, whereas only some H3.3-K27M tumours activate ALT. These findings suggest that acquiring the gly34 mutation on H3.3 might suffice to trigger the ALT mechanism.

**Supplementary Information:**

The online version of this article (10.1007/s00381-020-04933-8) contains supplementary material, which is available to authorized users.

## Introduction

Gliomas account for ~ 11% of all central nervous system (CNS) tumours in children aged 0–14 years. Paediatric high-grade glioma (pHGG) is one of the most common causes of cancer-related death under the age of 19 [42]. Despite histological similarities between pHGG and adult malignant gliomas, the former are more widely distributed within the CNS, with approximately 50% of cases occurring in midline locations [24].

The recent extensive use of high-throughput molecular, genetic and epigenetic profiling has considerably increased our knowledge of the cellular origin, pathogenesis and biological features of pHGG. This has helped to classify these neoplasms by their molecular/genetic features, which correlate with age of onset, anatomical location and prognosis [5, 11, 35, 40, 45, 51].

The maintenance of telomere length is a major molecular factor in human cancer development. Unlimited replication and immortalization are key features of neoplastic cells, needed to escape replicative senescence due to telomere shortening [41, 47]. Telomere length can be maintained via two different mechanisms: (i) reactivation of telomerase reverse transcriptase (TERT) via promoter mutations or methylation; and (ii) a telomerase-independent mechanism known as alternative lengthening of telomeres (ALT), which relies on the homologous recombination of telomeric regions [1, 2, 5, 19, 20, 26, 38], and results in a heterogeneous length and sequence composition [19, 20, 26].

Compared with adult HGG, pHGG differ in the frequency of isocitrate dehydrogenase (*IDH1/2*) mutations (< 6%, mostly in adolescents) [8, 35, 57], and telomerase reverse transcriptase promoter (*TERTp*) mutations (5 vs. 30–50% in adults) [11, 23, 26, 30, 35]. Hotspot mutations in histone H3.3 encoding gene (*H3F3A*) have also been found in > 50% of pHGG [35, 48], whereas they are rare in adult gliomas. With respect to this latter biomarker, the H3.3-K27M hotspot mutation is a characteristic of the “diffuse midline glioma (DMG) H3-K27M-mutant” [35, 51]. The clinico-pathological and biological significance of H3.3-G34R/V has yet to be fully elucidated [55], but this mutation will probably identify a separate biological entity in upcoming classifications.

The most common alterations related to telomerase upregulation are hotspot-activating mutations on *TERTp* C228T and C250T [5, 12, 25, 26, 30, 39]. Hyper-methylation of *TERTp* on the CpG-rich region − 600 bp upstream from the transcription start site (UTSS), which has been identified in paediatric brain tumours, may increase telomerase expression [4, 30]. *TERT* amplification and rearrangement are virtually absent in pHGG [2].

Tumours that do not activate telomerase may trigger the ALT phenotype. ALT is a recombination-based mechanism, where one telomere uses other chromosomal or extra-chromosomal telomeric DNA sequences as a template for telomere elongation [14, 20, 36, 38]. There are several hallmarks of the telomere length maintenance via ALT, including the following: (i) a long and heterogeneous telomere length [14, 19, 20, 54]; (ii) the presence of ALT-associated promyelocytic leukaemia (PML) nuclear bodies (APBs) [28, 54]; and (iii) the generation of high levels of C-rich circular telomeric DNA repeats (C-circles) [6, 22].

In pHGG, ALT is frequently activated by alterations of α-thalassaemia/mental retardation syndrome X-linked (*ATRX*) gene or, less frequently, in the *H3F3A* gene [12, 13, 18–20, 35, 46]. Loss of ATRX has been associated directly with extensive genome rearrangement, defective double-strand break repair and telomere elongation [3, 9, 33, 46], representing a molecular surrogate for the ALT phenotype [3, 25]. In adult HGG, inactivating mutations in *ATRX* and activating mutations in the *TERTp* are mutually exclusive, providing genetic evidence of ATRX loss contributing to telomere maintenance via the ALT phenotype in this cancer subtype [25].

A few studies have explored how *TERTp*, ATRX, H3.3-G34R/V and H3.3-K27M mutations are linked to activation of either of the two telomere maintenance mechanisms [12, 19, 46, 48, 49], but an assessment of telomere length in each molecular subgroup of pHGG is still lacking.

Hence, our present investigation study the relationships between ALT and molecular alteration in pHGG. We hypothesised that histone-defined pHGG subgroups could differ in terms of telomeric profile and ALT activation. To this end, we screened a cohort of IDH-wildtype, 1p/19q-wildtype pHGG, including H3.3-mutated, ATRX-lacking, *TERTp-*mutated and H3.3/ATRX/*TERTp*-wildtype cases for presence of the ALT phenotype. To do so, we used telomere-specific quantitative FISH on formal-fixed and paraffin-embedded (FFPE) samples, identifying any ultra-bright Tel-FISH signals [19, 20, 27, 37] . Since IDH mutations have been found in 35% of HGG patients over 14 years old 

## Materials and methods

### Study cohort and DNA extraction

The study was carried out on FFPE tissue specimens from pHGG collected at the national reference centre for paediatric brain tumours at “Sapienza Università di Roma”.

Tumours were classified by two neuropathologists (FG, MA) according to the World Health Organisation (WHO) classification of CNS tumours (2016), using standard histological and immunohistochemical methods (IHC) [32, 51]. Standard markers including GFAP, Ki67, IDH1-R132H and p53 were examined using IHC. As mentioned previously, all cases were negative for IDH1-R132H, as expected [8, 35, 43].

All FFPE tumours containing at least 70% of neoplastic cells were selected for DNA extraction with QIAamp DNA Mini Tissue Kit (Qiagen) according to the manufacturer’s instructions. DNA sequencing of *IDH1/2* genes, on exon 4, was done to rule out any point mutations in specimens from patients over 12 years old. [43]. FISH 1p-19q co-deletion was set to exclude oligodendroglial tumours (see Fig. 1 in the Supplementary material).

Informed consent was obtained from all participants or their parents before their inclusion in the study.

### ATRX nuclear loss, mutant H3-K27M and -G34R/V IHC

IHC analyses were performed on 3-μm sections of FFPE samples, using an automated immunohistochemical stainer (Leica Bond III). At least 1000 tumour cells were analysed, adopting the following scores: no expression = 0; weak expression = 1+; moderate expression = 2+; strong expression = 3 +.

Loss of ATRX nuclear expression was assessed by IHC because the presence of inactivating mutations on the ATRX gene results in loss of protein nuclear expression. As previously reported, loss of ATRX nuclear immunopositivity is widely considered a surrogate for *ATRX-*inactivating mutation status [9, 13, 25, 48]. ATRX (1:1000, NBP1-83077, Novus Biologicals) was scored as positive when neoplastic cells showed a staining intensity > 1, and cases with ≤ 15% immunopositive tumour nuclei were assumed to harbour ATRX-inactivating mutations. Endothelial cells were used for internal positive control purposes.

Selected samples (*n* = 18) were analysed using IHC with specific antibodies against H3.3 mutations. Commercially available antibodies were used to identify K27M (0.01 μg/mL, RM192, RevMAb Biosciences), G34R (1:400, RM240, RevMAb Biosciences) and G34V (1:4000, RM307, RevMAb Biosciences) mutant proteins [53].

Staining for H3.3K27M, -G34R and -G34V were scored as positive when > 15% of neoplastic cells showed nuclear immunoreactivity, associated with the negativity of internal control (endothelial cell nuclei) [53]. H3.3 status was confirmed with *H3F3A* sequencing on all 18 samples.

### *H3F3A* and *TERTp* hotspot mutation pyrosequencing

All samples were analysed using pyrosequencing to detect *H3F3A*; *TERTp* mutations were analysed via pyrosequencing for those cases where DNA was available (37/52). PCR primers were designed to amplify the *TERTp* region containing the C228T and C250T hotspots, corresponding to positions 124 and 146 bp upstream from the ATG site. Primers for the *H3F3A* mutational analysis were designed to amplify the hotspot codons on exon 2, corresponding to amino acids K27 and G34.

Pyrosequencing analysis was performed immobilising single-stranded DNA templates on streptavidin-coated sepharose high-performance beads (GE Health Care) using the PSQ Vacuum Prep Tool and Vacuum Prep Worktable. Sequencing was done using PyroGold reagents on the PyroMark Q24 instrument (Biotage), according to the manufacturer’s instructions. Negative controls were used to detect background signals. The pyrograms were analysed with the Pyro Mark Q24 software (Biotage) to ascertain the proportion of mutant vs. wild-type alleles according to the percentage relative peak height, as explained elsewhere [18]. All primers, PCR and sequencing conditions were as described in a previous study [37].

### *TERTp* methylation status

UTSS methylation status on *TERTp* was analysed using methylation-specific PCR (see Fig. 2 in the Supplementary materials). A semi-quantitative methylation-specific polymerase chain reaction (MS-PCR) was used to establish the methylation status of five CpG sites – 600 bp upstream from the *TERT* transcription start site (UTSS region). Bisulphite modification of DNA was conducted using the EZ DNA Methylation Kit (ZYMO Research), according to the manufacturer’s instructions. The five CpG sites were targeted using specific primers to amplify bisulphite-modified DNA. Two pairs of forward primers and one reverse primer, specific for methylated and unmethylated alleles, respectively (Supplementary Table 1), were used. Details of the PCR, cycling condition and analysis with ImageJ Software (NIH) are provided in a previous work [37].

### Telomere-specific fluorescence in situ hybridization and image analysis

Telomere length was assessed on 5-μm FFPE sections using telomere-specific fluorescence in situ hybridization (Tel-FISH). Telomere length was analysed with FITC-PNA probes (K532511, Dako) complementary to the telomeric-repeated sequences, according to the manufacturer’s instructions. The PNA probes do not recognise sub-telomeric sequences, enabling a precise measurement of telomere length (Dako). FISH sections were examined with an AxioImager M1 microscope (Carl Zeiss) by two investigators (SM and FRB). Signals were counted for 200 tumour cells, as reported in the literature [19, 20, 37]. ALT-positive cases were identified when ≥ 5% of tumour cells exhibited large, very bright intranuclear foci of Tel-FISH signals. Endothelial cell nuclei were used for normal internal control purposes.

Selected images were converted into TIFF files and exported for telomere-specific image analysis using the TFL-Telo V2 software (BC Cancer Research Centre, Vancouver) [44]. As previously reported [37], we used TFL-Telo to ascertain the number of telomeres, the fluorescence signal intensities and the distribution of telomeres in neoplastic cells for each case. The mean intensity of telomeres, assumed to be proportional to their total length, was measured for each sample on ≥ 100 neoplastic nuclei [37]. Other types of cell (e.g. infiltrating lymphocytes, endothelial cells) were excluded from the digital image analysis based on their morphological features.

The telomere fluorescence threshold (mean intensity ≈ 1000) was established by using positive and negative controls (previously analysed brain tumours with ALT activation and non-neoplastic brain tissues or telomerase/ALT negative gliomas, respectively). For comparison, we analysed 1 pilocytic astrocytoma (AP; WHO grade I), 1 primitive neuroectodermal tumour (PNET), 1 gliosarcoma (GSM, grade IV) and 1 oligoastrocytoma (OA; WHO grade II) (see Supplementary Table 1). Different telomeric profiles were identified in our cases (as shown Fig. 3). TFL-Telo analysis revealed significant differences in telomere length (*p* < 0.0001) between the positive and negative controls.

### Statistical analysis

All data were recorded using Microsoft Excel 2016 and analysed using MedCalc statistical software (MedCalc Software Ltd, Belgium). A multifactor analysis of variance (ANOVA) was used to compare two or more groups. A *p* value < 0.05 was considered statistically significant. The correlation between genetic changes and patients’ age was tested using linear regression and the *t* test assuming unequal variances.

## Results

Patients’ demographic and clinical features are summarised in Table 1. We analysed 52 cases of pHGG (17 females and 35 males; age range 0.3 to 24 years, median 12.1 years). Based on the patients’ neuropathological features, the sample included 32 glioblastomas (GBM, WHO grade IV), 10 K27-mutant diffuse midline gliomas (DMG, WHO grade IV), 5 high-grade astrocytomas (AA, WHO grade III) and 5 high-grade glioneuronal tumours (GNT). As previously stated, all cases included in this study were negative for IDH1-R132H. As expected [43], none of the patients over 12 years old showed any IDH1/2 gene point mutations apart from a biomolecular resemblance to malignant gliomas arising in > 14-year olds and adults. All recruited cases had normal 1p/1q and 19q/19p, thereby excluding oligodendrogliomas.

**Table 1:** Summary of demographic and clinico-pathological information of pHGG series

	Features	Frequency (*n* = 52 pHGG)
Sex	Male	67.3% (35/52)
	Female	32.7% (17/52)
Age at diagnosis	≤ 5 years	17.3% (9/52)
	6–10 years	25% (13/52)
	10–15 years	17.3% (9/52)
	15–24 years	40.4% (21/52)
	Mean = 12.1 years	
Location	Supratentorial	84.6% (44/52)
	Sub-/infratentorial	15.4% (8/52)
Histology	Glioblastoma	61.6% (32/52)
	Diffuse midline glioma	19.2% (10/52)
	Anaplastic astrocytoma	9.6% (5/52)
	High-grade glioneuronal tumour	9.6% (5/52)

We analysed the gene alterations involved in maintaining telomere length, such as *TERTp*, *H3F3A* and ATRX. We examined telomere length using a robust Tel-FISH assay to identify the activation of ALT. We measured telomere length on each sample using image analysis software, and correlated the presence of each specific mutation with the telomere length profile.

The detailed list of 52 samples, the tests performed on each sample and the results are provided in Table 1 in the Supplementary material.

### Genetic alterations involved in telomere maintenance mechanisms

**Fig. 1:**
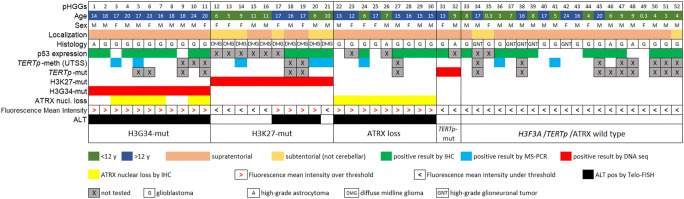
Relevant clinico-pathological, genetic and molecular features of pHGG in our cohort, showing the increased telomere fluorescence intensity and ALT activation in different molecular subgroups of cases with H3G34R mutations, H3K27M mutations, ATRX nuclear loss with H3.3-wt, *TERTp* mutations or no alterations on these genes

*H3F3A* mutations were found in 21/52 cases (40%). There were 10/52 (19%) K27M mutations and 11/52 (21%) G34R mutations. None of the samples analysed showed G34V mutation or other *H3F3A* variants (0%). ATRX nuclear loss was apparent in 16/52 (31%) cases. Loss of ATRX expression was associated with the G34R mutation in 7/16 cases, but in none of the K27M-mutated or *TERTp*-mutated samples. The results are summarised in Table 2. Genetic and molecular features, histological and clinical characteristics, and telomere length results are shown in Fig. 1.

**Table 2:** Summary of results for ATRX, H3G34R, H3K27M and *TERTp* mutations, *TERTp* methylation and telomere elongation. The incidence of any alteration is reported for the whole cohort of pHGG and stratified by histology

Tumour type	ATRX nuclear loss	H3G34R-mut	H3K27M-mut	*TERTp*-mut	*TERTp*-meth	Telomere elongation
pHGG (*n* = 52)	30.7% (16/52)	21.1% (11/52)	19.2% (10/52)	5.4% (2/37)	21% (8/38)	46.1% (24/52)
GBM (*n* = 32)	46.8% (15/32)	31.2% (10/32)	0%	3.1% (1/32)	21.7% (5/23)	56.2% (18/32)
DMG (*n* = 10)	0%	0%	100% (10/10)	0%	37.5% (3/8)	40% (4/10)
High-grade A (*n* = 5)	20% (1/5)	20% (1/5)	0%	20% (1/5)	0%	40% (2/5)
High-grade GNT (*n* = 5)	0%	0%	0%	0%	0%	0%

*TERTp* mutations were rare. The C250T mutation was found in 2/37 cases (5.4%), while hyper-methylation of *TERTp* on UTSS was detected in 8/38 cases (21%). Among these 8 UTSS hyper-methylated cases, 2 were H3.3/TERT/ATRX-wildtype, 1 was associated with ATRX nuclear loss, 3/8 with the K27M mutation and 2 with the G34R mutation. Representative results regarding the *H3F3A* and *TERTp* mutations, ATRX nuclear expression and UTSS methylation status on *TERTp* are shown in Fig. 2 in the Supplementary material.

G34R and K27M mutations were mutually exclusive and were never associated with *TERTp* mutations, as previously reported [35, 48, 55]. H3.3-G34R-mutant tumours were all supratentorial and associated with ATRX nuclear loss in 7/11 cases (64%). On the other hand, cases with the K27M mutation (midline) were never associated with loss of ATRX nuclear expression. Eventually, patients with G34R-mutant pHGG had a significantly higher median age at diagnosis (*p* = 0.0014) than those with the K27M mutant (18.6 vs. 10.8 years) (Fig. 3 in the Supplementary materials), a finding consistent with previous reports [37, 55]. The frequency of cases with alterations varied by histopathological variant (Table 2).

ATRX nuclear loss was more common in GBM (47%). H3.3-G34R variant was detected mainly in GBM (31%) and less frequently in high-grade astrocytomas (20%), while H3.3-K27M variant was found exclusively in DMG (WHO). *TERTp* hyper-methylation was only seen in 22% of GBM and 37% of DMG.

### ALT is frequently activated in pHGG

**Fig. 2:**
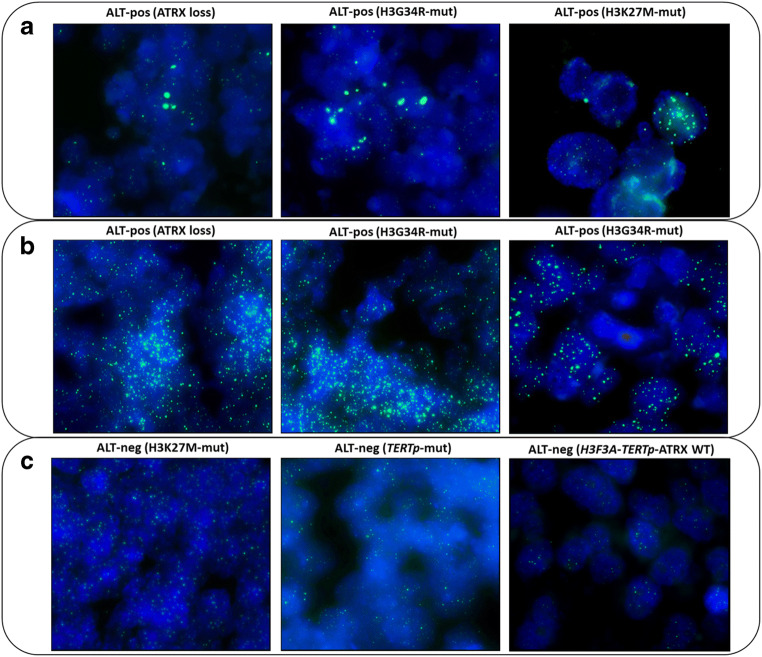
Panel of representative cases analysed for telomere elongation using Tel-FISH (magnification × 100). Green signals represent telomeres. **a** pHGG with large, very bright intranuclear foci of telomere signals in single cells. **b** Cases with telomere elongation featuring very bright signals in large neoplastic regions. **c** Cases with small and homogeneous telomere foci, indicative of basal-level telomere length and no ALT

pHGG were analysed using Tel-FISH to assess telomere length (Fig. 2). Telomere elongation due to the ALT mechanism was seen in 24/52 cases (46%), with a significant increase in the intensity of telomere fluorescence. In our cohort, ALT activation was associated exclusively with *H3F3A* or ATRX alterations. Three different ALT activation profiles were identified: pHGG with single tumour cells featuring large and very bright intranuclear telomere signals (≥ 5% of cells); pHGG with homogeneous areas of telomere elongation characterised by bright signals in large neoplastic regions; and pHGG with small, homogeneous telomere signals indicative of a basal-level telomere length. It is worth noting that the 2 cases carrying *TERTp* mutations were not associated with any increase in telomere length (Fig. 2), whereas 6/8 *TERTp* hyper-methylated pHGG showed telomere elongation via ALT, promoted by *H3F3A* mutations or ATRX loss. ALT was detected in GBM (56.2%), DMG (40%) and high-grade astrocytomas (40%). There was no increase in telomere length in GNT or pilocytic astrocytomas, PNET, gliosarcomas or oligoastrocytomas used for control purposes.

### H3.3 G34R and K27M mutations show different activation of ALT

**Fig. 3:**
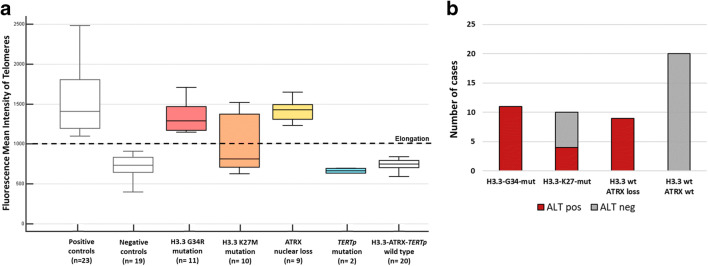
Telomere length assessed in 52 pHGG. **a** Telomere fluorescence intensity quantified in different subgroups with H3.3-G34R mutations (red), H3.3-K27M mutations (orange), ATRX nuclear loss (yellow), *TERT* promoter mutations (light blue) or no alterations on these genes; positive and negative controls (grey boxes) were included to establish a threshold (dotted black line). **b** Number of cases with H3.3-G34-mut, H3.3-K27-mut, H3.3-wt with ATRX loss and H3.3/ATRX-wt, stratified according to ALT activation

We analysed the relationships between telomere length and ATRX, H3G34, H3K27 and *TERTp* alterations (Fig. 3a), measuring telomere length with Tel-FISH and using TFL-Telo image analysis software, as described previously [37].

Telomere elongation was found in cases with *H3F3A* mutations. Interestingly, all (11/11) G34R-mut pHGG showed increased telomere length (Fig. 3b). Cases with H3.3-G34R variant showed ALT activation even in the absence of ATRX nuclear loss. Conversely, only 4/10 cases with the K27M mutation (40%) had elongated telomeres, while the other 6/10, 60%) showed a telomere length comparable with that of negative controls. These results suggest that lysine27 mutation is not sufficient to trigger ALT (Fig. 3a–b). Notably, all H3.3-K27M-mutated pHGG showed a retained ATRX protein expression, as if ALT occurred irrespective of ATRX status. Telomere elongation was also found in all 9/9 cases with ATRX nuclear loss (100%) (Fig. 3), always correlating with ALT activation irrespective of *H3F3A* status. Telomere length was within control range in pHGG associated with TERTp mutations, possibly as a consequence of the lack of concurrent hyper-methylation of the TERT promoter on the UTSS region. Cases with no ATRX-*H3F3A-TERTp* alterations exhibited a normal telomere length (Fig. 3a).

Differences in the mean intensity of telomere fluorescence between the G34R- and K27M-mutant molecular subgroups were statistically significant (*p* < 0.05).

## Discussion and conclusion

Maintenance of telomere length is a fundamental process by which cancer cells escape replicative senescence. Two different pathways sustain this process: reactivation of telomerase via *TERT* promoter mutations or hyper-methylation; and the ALT mechanism triggered by alterations on *ATRX* or *H3F3A* [1, 2, 5, 14, 19, 20, 26, 36, 38, 46]. In adult HGG, inactivating mutations in *ATRX* and *TERTp* are mutually exclusive, indicating that *ATRX* loss contributes to telomere maintenance via the ALT phenotype. As to the association between *H3F3A* mutations and ALT, a previous study showed that H3.3-G34R/V mutations were associated with global DNA hypo-methylation at the end of chromosomes. Neoplasms with G34R/V mutations have a significant overlap with those involving *ATRX* mutations and there is evidence of tumours with the H3.3-K27M mutation using ALT.

Our results show that pHGG frequently acquire a telomere maintenance mechanism via the ALT pathway (46%), while telomerase reactivation via *TERTp* mutations/hyper-methylation is not associated with telomere elongation. This finding demonstrates that ALT is the main telomere elongation pathway in pHGG, unlike the case of adult HGG, which frequently shows telomerase reactivation triggered by *TERTp* mutations (30–50%) [5, 11, 30]. The literature shows that *TERTp* mutations are extremely rare in paediatric tumours (< 5%) [2, 11, 23, 26, 30, 35].

Previous data showed that cancer cells with reactivation of telomerase preferentially target short telomeres [1, 21] compared with normal samples [2, 50]. A previous study showed that *TERTp* mutations do not activate telomerase expression enough to counteract telomere shortening [7], and this gives rise to cells with critically short and unprotected telomeres. Our results highlight that telomeres are not elongated in pHGG harbouring *TERTp* mutations, supporting that such mutations do not lead to any notable telomere elongation detected by quantitative FISH. A previous study on DNA methylation of the TERT promoter showed that UTSS hyper-methylation was associated with tumour progression and a poor prognosis [4, 30, 37]. The present findings suggest that UTSS hyper-methylation alone (2/8) is not sufficient to activate telomere elongation, as well as a *TERTp* mutation. This would mean that the maintenance of telomere length through telomerase reactivation requires a gradual upregulation of telomerase promoted by multiple steps [7]. Since UTSS hyper-methylation is only associated with telomere elongation and ultra-bright intranuclear telomere foci when concomitant H3.3 or ATRX alterations are present, therefore, telomere elongation must be driven by ALT pathway activation in our cases.

The present findings show a strong association between H3.3 mutations and ALT. Thus, all our G34R-mutant pHGG showed the ALT phenotype, while the K27M-mutant cases exhibited a much lower incidence of ALT activation (40%). Interestingly, the G34R-mutant subgroup always used ALT to maintain telomere length, with either a simultaneous ATRX nuclear loss (63.6%) or a retained ATRX (36.4%). This finding suggests that the acquisition of a H3.3-glycine34 mutation might per se trigger the ALT mechanism, regardless of ATRX status. On the other hand, only a subset of H3.3-K27M-mutant gliomas triggered ALT, while the majority of cases (60%) showed telomere shortening.

There were no differences as to *TERTp* or ATRX status between the K27M-mutant pHGG that activated ALT and those that did not, suggesting that other concomitant mutations or factors might contribute to activating ALT in such cases. As expected, cases with ATRX nuclear loss always trigger ALT, regardless of *H3F3A* status, confirming a strong association with ALT in pHGG [2, 9, 25, 46]. Moreover, our results also demonstrate that *H3F3A*/ATRX/*TERTp*-wildtype pHGG never activate mechanisms for telomere elongation (this was seen in none of our 20 cases).

Despite this study’s limitations, including the small size of our cohort and the absence of outcome correlations, the present findings add to what we know about the differences in the molecular profiles between adult HGG and pHGG. Our results provide preliminary evidence of differences in the incidence of telomere elongation in different molecular subgroups of pHGG, which may be relevant to the development of different therapeutic approaches to different pHGG populations. Research is currently underway on therapeutic strategies aimed at targeting molecules involved in telomere maintenance mechanisms. Early trials have produced encouraging results in adult HGG subtypes lacking histone H3.3 mutations, such as telomerase inhibitors for *TERTp*-mutant gliomas and ATR inhibitors for ALT-positive neoplasms with ATRX nuclear loss [23, 29]. If such ALT-targeted treatments will enter novel therapeutic protocols for molecular subgroups of pHGG, the analysis of telomere length would be necessary to pinpoint appropriate patients.

Potential ALT-targeted drugs have recently been proposed to hit heterochromatin formation driving the onset of ALT; the ATRX/DAXX/H3.3 complex, inhibitors of recombination factors (ATR, RAD52, SETDB1, FANCM) histone deacetylase inhibitors, G-quadruplex stabilisers; APB formation inhibitors; and other chromatin remodelling strategies [10, 15–17, 31, 34, 52, 56].

Our study revealed differences in the histone-defined subgroups of pHGG in terms of their telomeric profiles and ALT activation, improving our understanding of the association between histone mutations and ALT, and pointing to the possibility of using new targeted treatments for a subset of these tumours.

Although further validation on a larger prospective cohort will be necessary, our findings might contribute to the future use of highly selective therapeutic strategies for a subset of ALT-positive H3.3-mutated paediatric high-grade gliomas.

## Supplementary Information

ESM 1 (DOC 6.41 mb)

Supplementary Table 1 (XLS 40.5 kb)

High resolution image (TIF 6.00 mb)

Supplementary Fig. 1 (PNG 2.76 mb)

High resolution image (TIF 7.53 mb)

Supplementary Fig. 2 (PNG 1.19 mb)

High resolution image (TIF 5.60 mb)

Supplementary Fig. 3 (PNG 54.1 kb)

High resolution image (TIF 7.66 mb)

Supplementary Fig. 4 (PNG 879 kb)

## Data Availability

Data available on request due to privacy/ethical restrictions.
