# Supplementary material for: Alternative lengthening of telomeres in molecular subgroups of paediatric high-grade glioma
Source: Childs Nerv Syst. 2020 Oct 31;37(3):809–18. doi: 10.1007/s00381-020-04933-8 (PMC7875853; doi:10.1007/s00381-020-04933-8)
Supplement: Supplementary file 1 — (DOC 6.41 mb) [file 381_2020_4933_MOESM1_ESM.doc]

**Telomere maintenance mechanisms in molecular subgroups of Pediatric High-Grade Gliomas. Simone Minasi^1,2^, Caterina Baldi^2,3^, Francesca Gianno^1,3^, Manila Antonelli^1^, Anna Maria Buccoliero^4^, Torsten Pietsch^5^, Maura Massimino^6^, Francesca Romana Buttarelli^2,1*^.**

Corresponding author: Francesca Romana Buttarelli, [francesca.buttarelli@uniroma1.it](mailto:francesca.buttarelli@uniroma1.it)


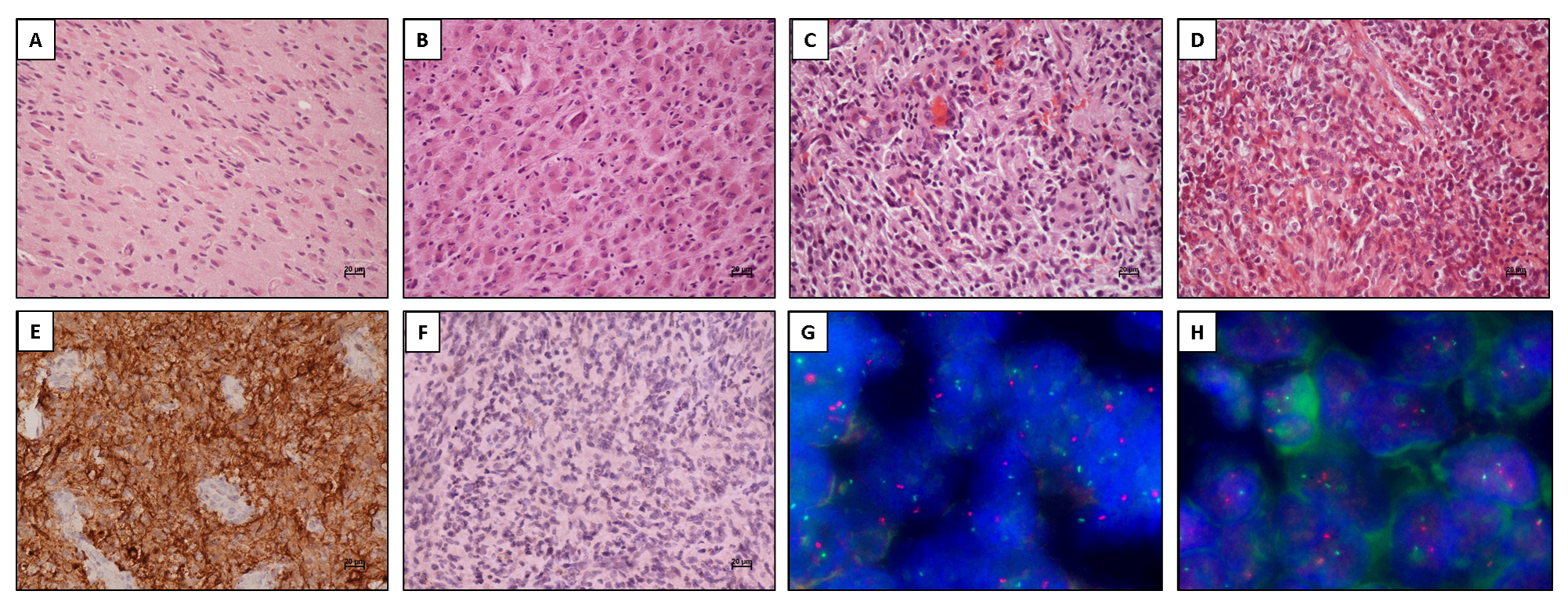


**Supplementary Fig. 1:** Panel of H&E (A, B, C, D) shows astrocytic tumor (A, B) and GBM (C, D) representative of our pHGG cohort. Immunohistochemical panel of a representative sample evidences immunopositivity for GFAP (E), with negative endothelial cells, and immuno-negativity for IDH1 R132H mutant (F). Representative images of Fluorescence in situ hybridization (G, H), using dual colour probes (Vysis LSI 1p36 Spectrum Orange and 1q25 Spectrum Green; Vysis LSI 19q13 Spectrum Orange and 19p13 Spectrum Green, Abbott Molecular) to determine the deletion status of chromosome arms 1p (G) and 19q (H), show tumor cells with normal 1p/19q copy number.


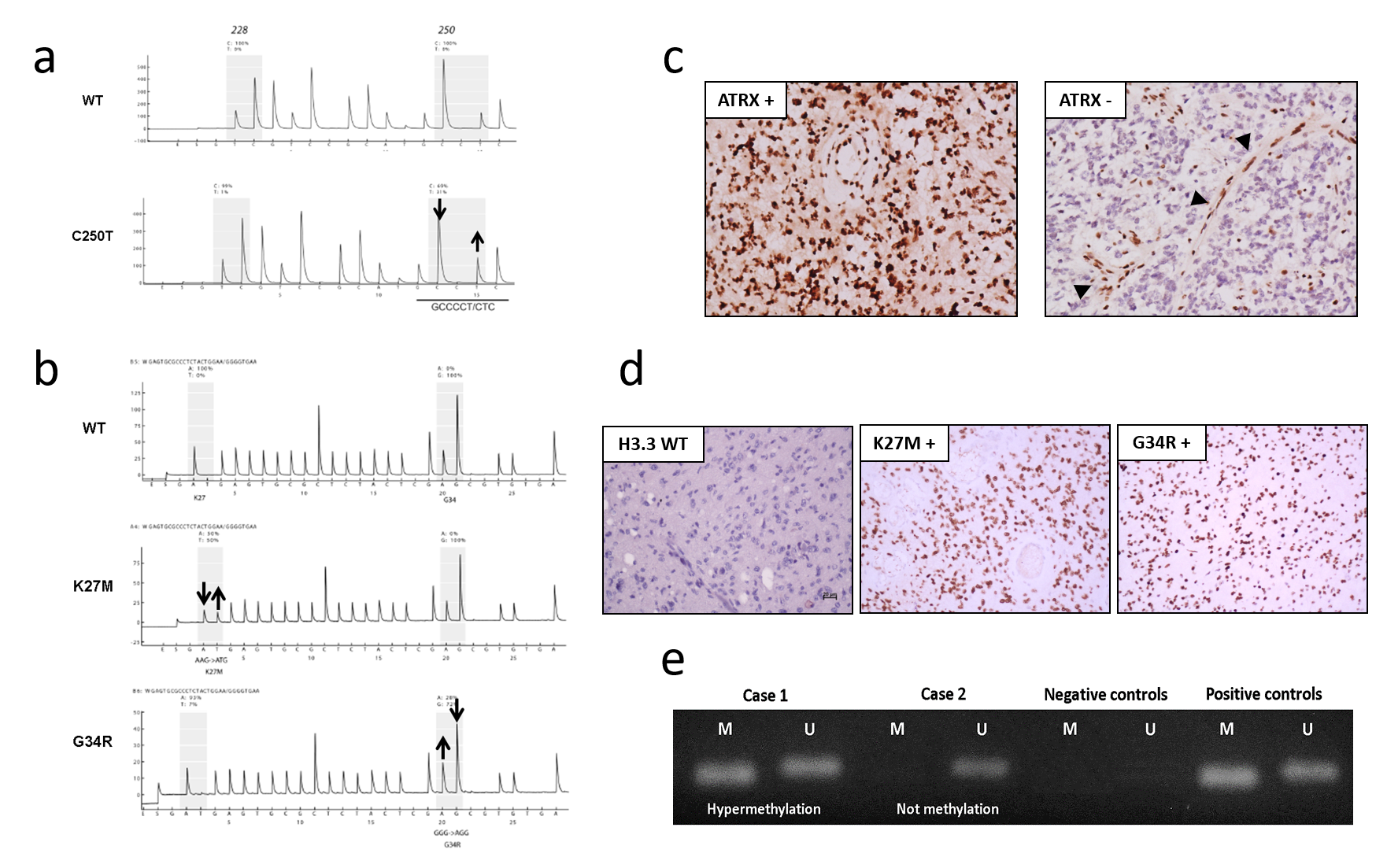


Supplementary Fig. 2: Representative results for *TERTp* (a) and *H3F3A* mutations (b) via pyrosequencing, ATRX nuclear loss (c) and H3K27M or H3G34R (d) via IHC, and UTSS methylation status (e) via MS-PCR.

(a) From the top: selected wild-type sample for *TERTp* mutations and selected sample with C250T mutation. (b) From the top: selected wild-type sample for *H3F3A* mutations, case with H3K27M mutation and case with H3G34R mutation (arrows indicate proportional variations in the high of the peaks that evidence the presence of a specific point mutation). (c) Selected sample with 100% nuclear immuno-positivity for ATRX and selected sample with nuclear immuno-negativity >60% of neoplastic cells for ATRX (magnification 40X); arrows indicate endothelial cells used as internal positive control. (d) Few samples were also analyzed via IHC for H3K27M and H3G34R/V mutations. Images show selected H3.3 wild-type sample with 100% nuclear immuno-negativity, and selected H3.3 mutated samples with nuclear positivity for K27M and G34R, with negative endothelial cells; all cases were then confirmed by sequencing. (e) Selected samples with UTSS region hyper-methylated and un-methylated via MS-PCR; positive and negative controls were included.


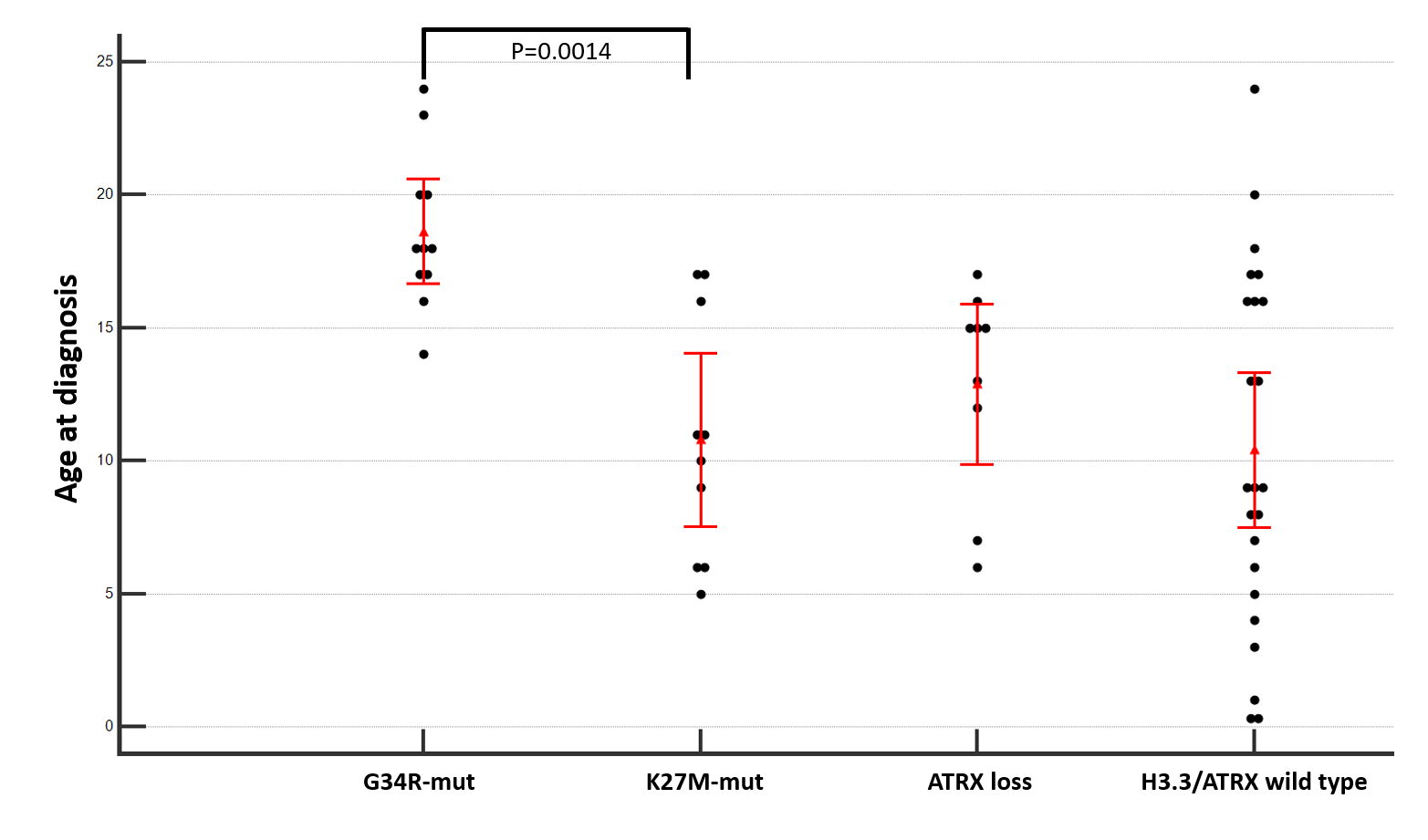


**Supplementary Fig. 3:** Age at diagnosis of 52 IDH-wt pHGGs and 4 control cases with pLGGs classified in 4 different molecular subgroups.

Presence of G34 mutations was associated with older age compared to K27-mut pHGGs (median age=17.9 vs. 10.8; P=0.0014, t test assuming un-equal variances). The only two cases with *TERTp* mutations, not included in figure, had 9 and 12 yrs. respectively, in line with median age of our cohort.


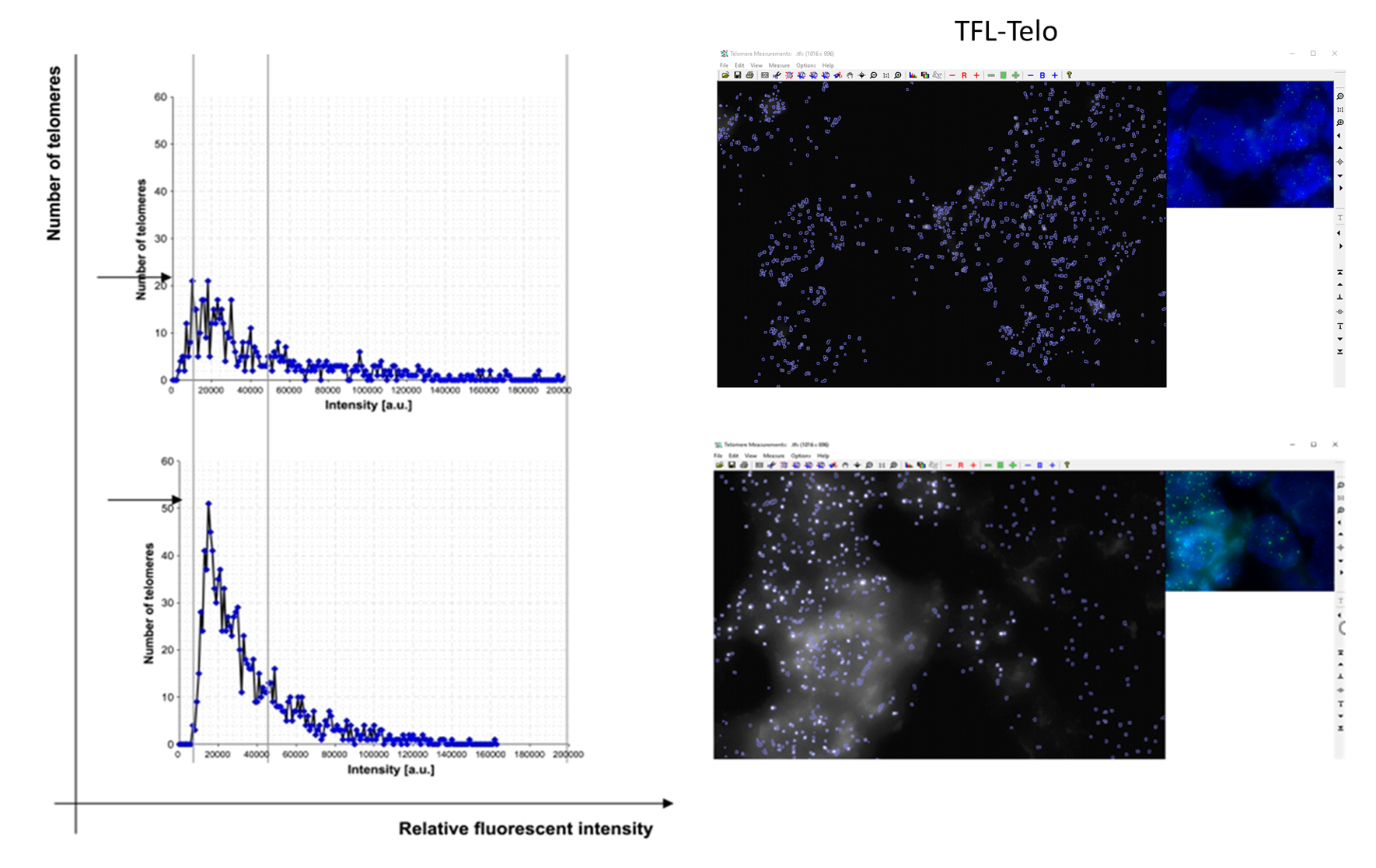


**Supplementary Fig. 4:** TFL-Telo is an application used to estimate the length of telomeres from captured images of cells that have been analyzed by FISH with florescence probes specific for telomeric sequence. This representative image shows how telomeres are highlighted within the nucleus; histograms show correlation between telomeres number and relative fluorescence intensity, to calculate the mean intensity of telomeres for each analyzed image. The histogram above is representative of an ALT negative case, while the histogram below shows the profile an ALT positive pHGG.
