# Supplementary figures and images for: Alternative lengthening of telomeres in molecular subgroups of paediatric high-grade glioma
Source: Childs Nerv Syst. 2020 Oct 31;37(3):809–18. doi: 10.1007/s00381-020-04933-8 (PMC7875853; doi:10.1007/s00381-020-04933-8)

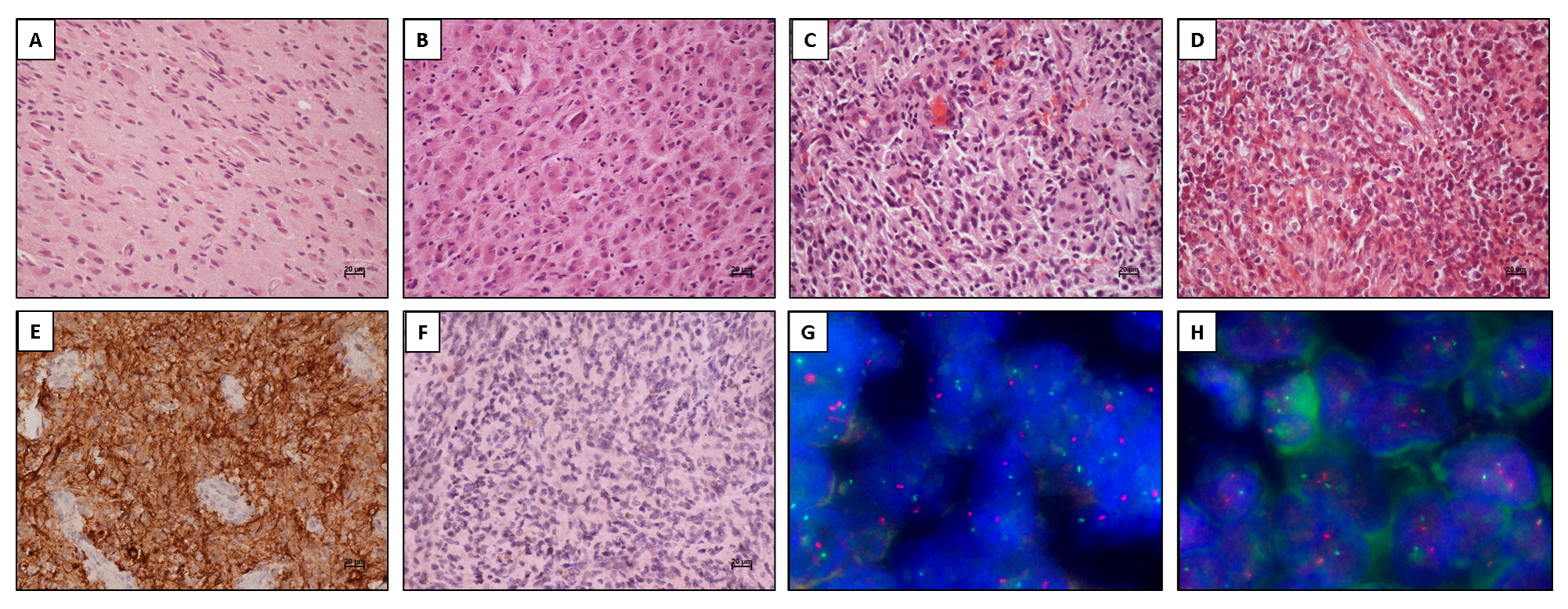

Supplement: Supplementary file 3 — High resolution image (TIF 6.00 mb) [file 381_2020_4933_MOESM3_ESM.tif]

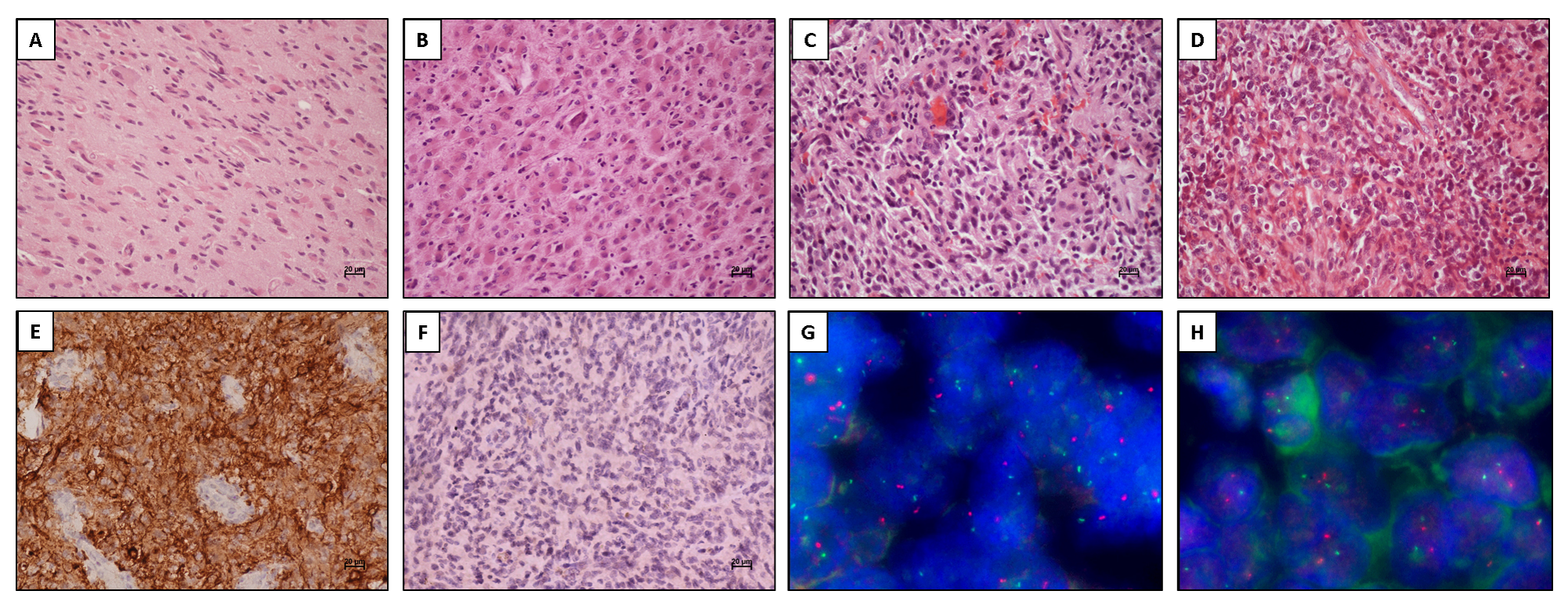

Supplement: Supplementary file 4 — (PNG 2.76 mb) [file 381_2020_4933_Fig4_ESM.png]

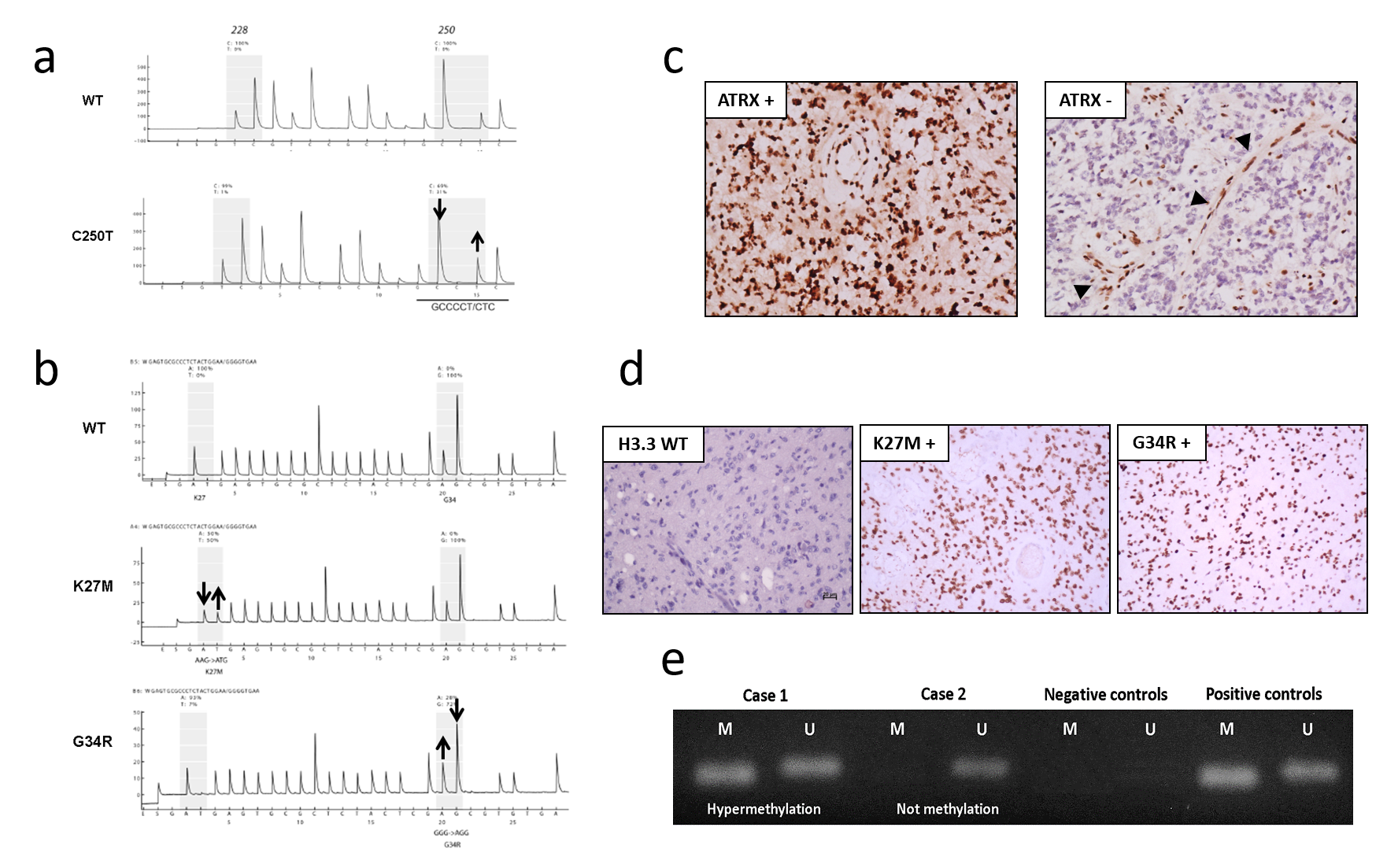

Supplement: Supplementary file 5 — High resolution image (TIF 7.53 mb) [file 381_2020_4933_MOESM4_ESM.tif]

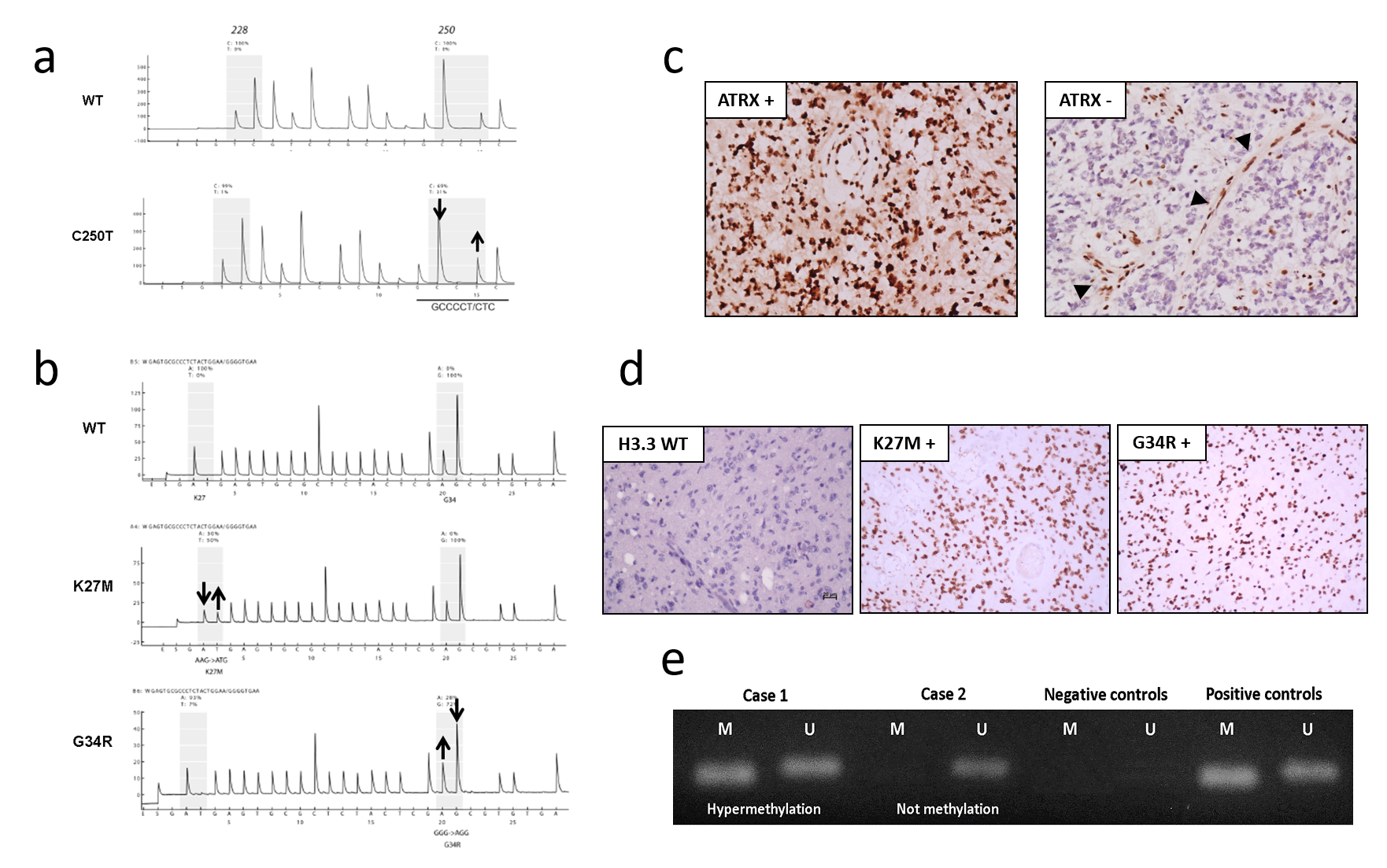

Supplement: Supplementary file 6 — (PNG 1.19 mb) [file 381_2020_4933_Fig5_ESM.png]

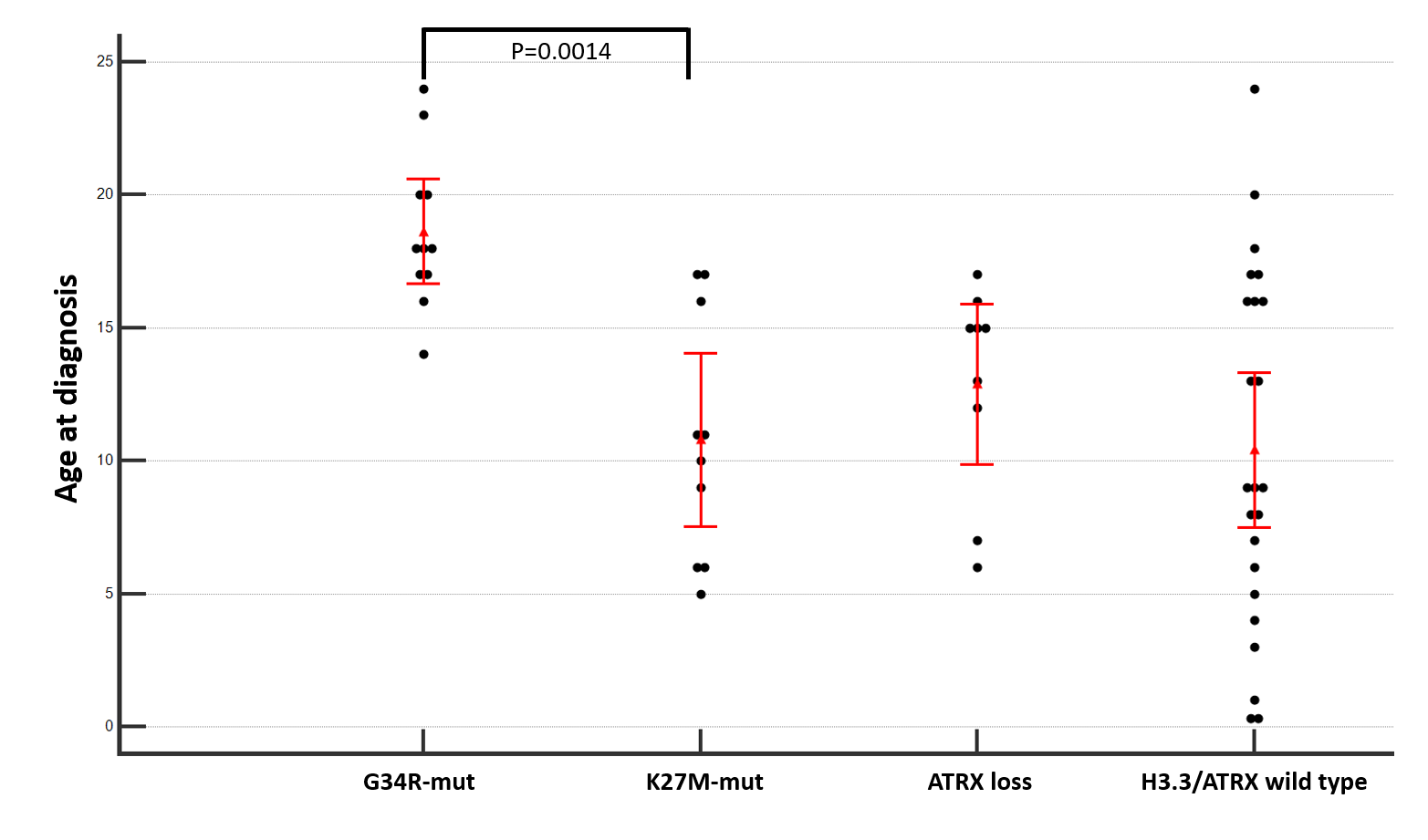

Supplement: Supplementary file 7 — High resolution image (TIF 5.60 mb) [file 381_2020_4933_MOESM5_ESM.tif]

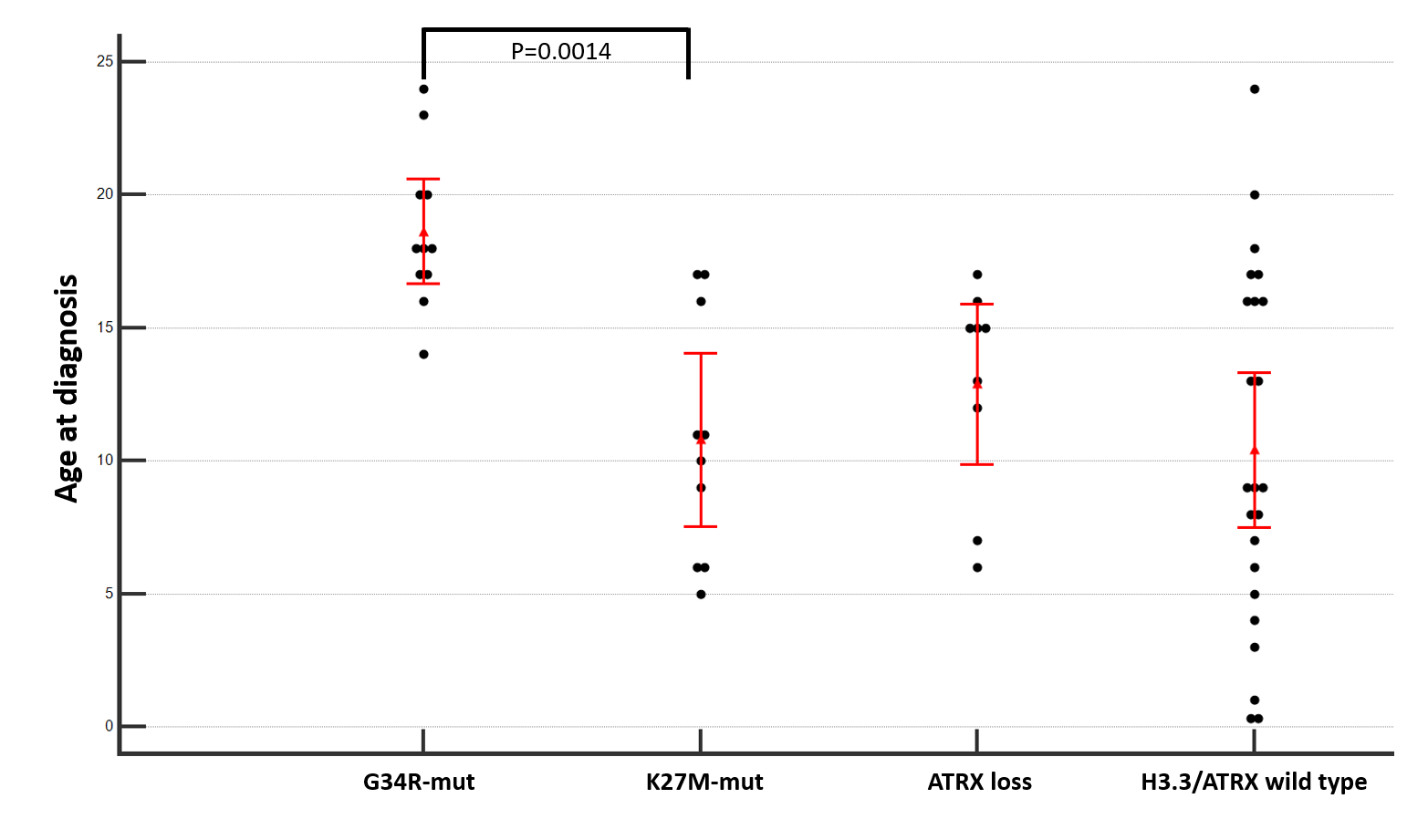

Supplement: Supplementary file 8 — (PNG 54.1 kb) [file 381_2020_4933_Fig6_ESM.png]

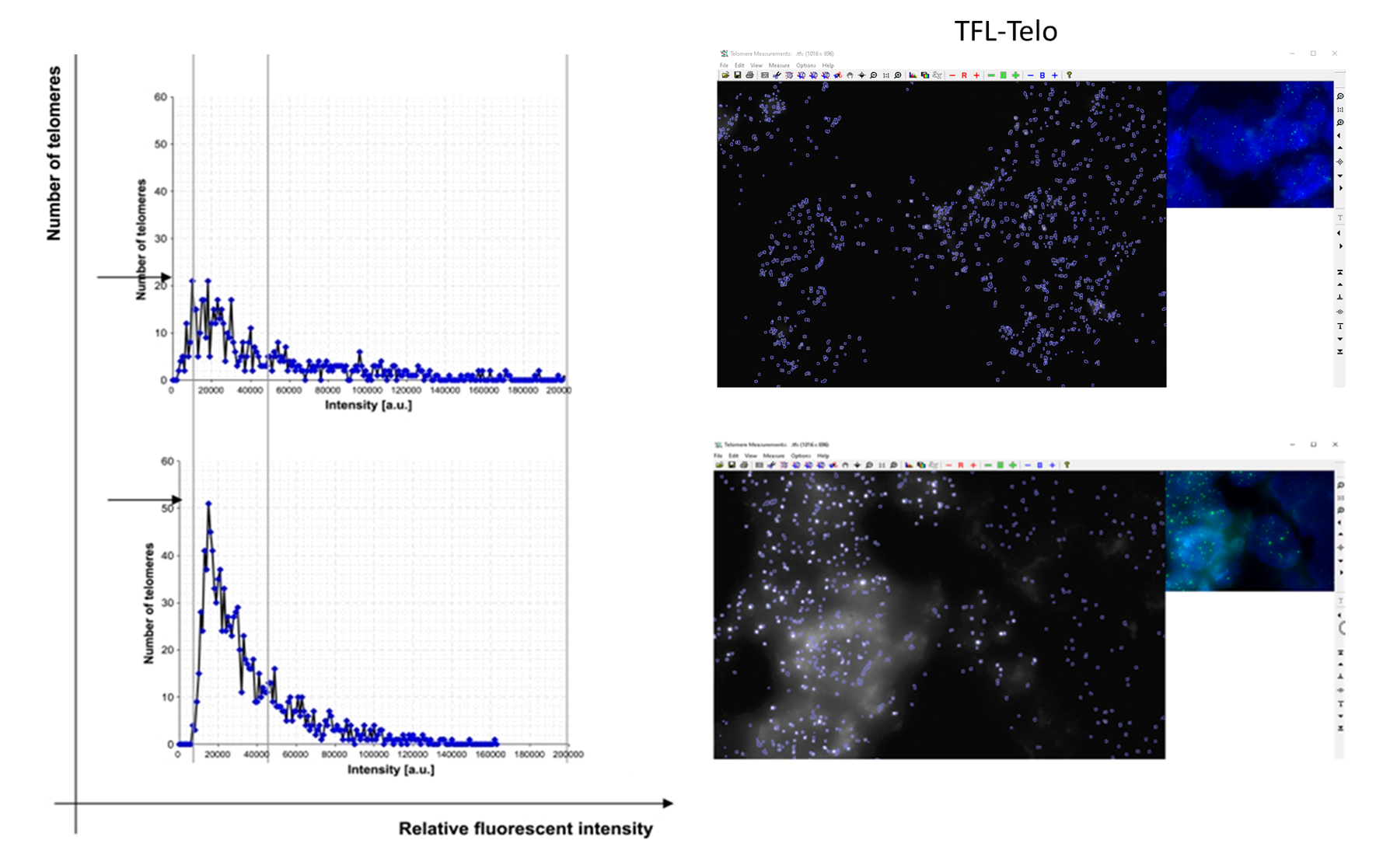

Supplement: Supplementary file 9 — High resolution image (TIF 7.66 mb) [file 381_2020_4933_MOESM6_ESM.tif]

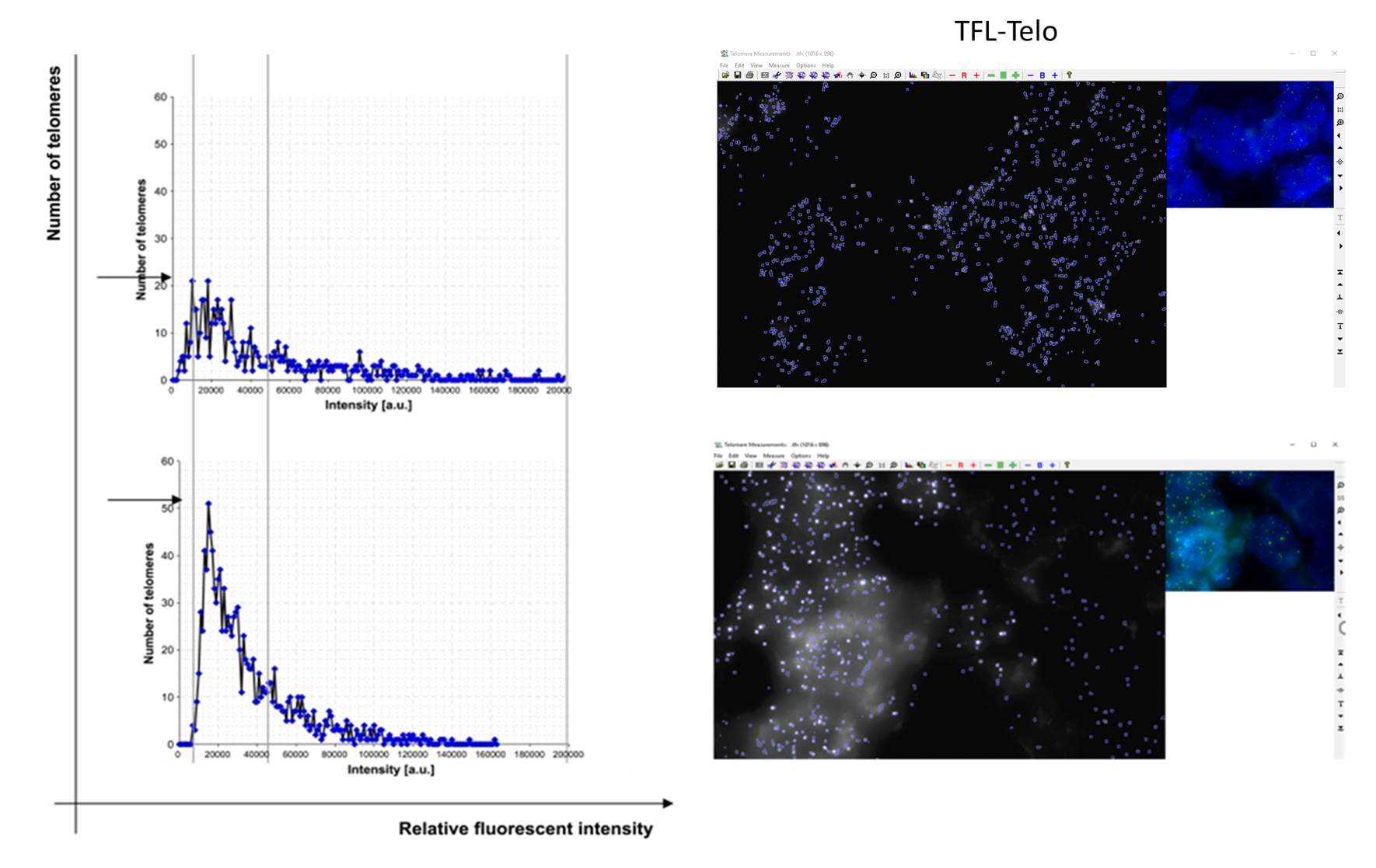

Supplement: Supplementary file 10 — (PNG 879 kb) [file 381_2020_4933_Fig7_ESM.png]
